# A Comparative Analysis on the Environmental Impact of Selected Methods for Determining the Profile of Fatty Acids in Cheese

**DOI:** 10.3390/molecules28134981

**Published:** 2023-06-25

**Authors:** Izabela Narloch, Grażyna Wejnerowska

**Affiliations:** Department of Food Analysis and Environmental Protection, Faculty of Chemical Technology and Engineering, Bydgoszcz University of Science and Technology, 85-326 Bydgoszcz, Poland; izabela.narloch@pbs.edu.pl

**Keywords:** dairy products, environmental assessment tools, food analysis, food composition, gas chromatography, greenness

## Abstract

The fatty acid profile of cheese influences its sensory parameters, such as color, texture, or flavor. Examining the fatty acid profile also helps to assess the nutritional value of the cheese that is being tested. However, the determination of fatty acids in cheese samples is a multi-stage and time-consuming task. In addition, large amounts of toxic organic solvents are used to prepare samples for analysis purposes. This paper presents the results of a study to determine the fatty acid profile of yellow cheese samples. Six different methods of sample preparation were compared for analysis purposes. The profile of fatty acids was determined using gas chromatography with flame ionization detection (GC-FID). The study showed significant differences (*p* > 0.05) in the resulting fatty acid profile between the methods used. It was found that the most reliable fatty acid profile results were obtained using methods derived from the Folch method. In addition, tools such as the Analytical Eco-Scale tool and the Analytical Greenness Metric for Sample Preparation (AGREEprep) tool were used to assess the greenness of the methods used. In the case of the Analytical Eco-Scale tool, all six methods scored ‘acceptable green analysis’ with scores ranging from 61 to 73. However, an evaluation of methods using the AGREEprep metric showed that the results of the methods (0.13–0.27) did not show the “greenness” of the analytical methods.

## 1. Introduction

Cheese is a nutrient-rich food source of health-promoting compounds in the human diet. Fatty acid (FA) analysis of cheese is important in determining its nutritional value (labelling). In addition, FA analysis helps to examine cheese technology and is used to study the influence of various factors on the FA profile in milk (diet and breed of animals, season, etc.) [1,2]. Cheese’s nutritional and sensory values are influenced by many factors, including milk characteristics, starter cultures, and technological processing [3,4]. Cheese characteristics shown are affected by the FA profile of milk used in its production. The degree of unsaturation of FA influences the texture of cheese. The higher the unsaturation, the softer the texture of the cheese. In the case of flavor, rancidity increases when the free FAs are released. Additionally, a pungent flavor is associated with a higher amount of short-chain FAs [5,6,7,8].

The complexity of the cheese matrix due to FA profile analysis requires the use of appropriate analytical procedures. The selection of appropriate methods plays a critical role in the efficient (qualitative and quantitative) extraction of major and minor lipids. Typically, these methods involve the use of large amounts of toxic solvents and therefore generate a lot of waste, and are laborious and time-consuming. Several evaluation methods allow the selection of the most efficient method for FA analysis in the cheese sample in terms of the greenness of analytical procedures. Several tools are employed in green analytical chemistry to study the environmental performance of an analytical procedure, including the Analytical Eco-Scale tool, the National Environmental Methods Index (NEMI), the Green Analytical Procedure Index (GAPI), the Analytical Greenness (AGREE) tool, and the newer Analytical Greenness Metric for Sample Preparation (AGREEprep) tool. Objective criteria related to analytical performance, environmental impact, sustainability, and economic cost are evaluated by these tools through the definition of penalty points [9,10,11,12,13,14,15,16]. This makes it possible to choose the most environmentally friendly analytical procedure, which is very important nowadays for both ecological and also economic and analytical reasons.

In the literature, there are several methods available for preparing cheese samples for FA profile studies, but none compare their impact on the test results. In order to confirm the reliability of the authors’ research, six methods were selected from those available in the literature to compare their results. In addition, these methods were evaluated using environmental assessment tools, which helped to evaluate the environmental impact of these procedures. The conclusions from this study are intended to draw the attention of researchers to analytical problems and ecological aspects when performing cheese analysis.

## 2. Results and Discussion

### 2.1. Optimization of GC-FID Conditions

**Figure 1:**
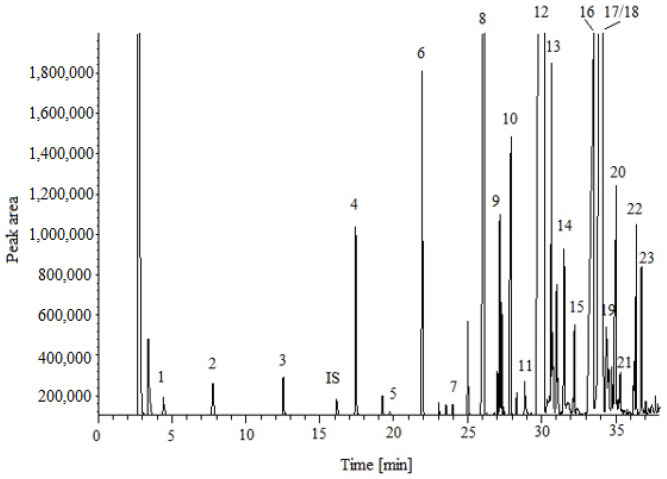
Exemplary chromatogram of cheese lipid FAMEs (the sample was prepared in accordance with method C). The peaks are: 1. butyric acid, 2. caproic acid, 3. caprylic acid, 4. capric acid, 5. undecanoic acid, 6. lauric acid, 7. tridecanoic acid, 8. myristic acid, 9. myristoleic acid, 10. pentadecanoic acid, 11. cis-100-pentadecanoic acid, 12. palmitic acid, 13. palmitoleic acid, 14. heptadecanoic acid, 15. cis-heptadecanoic acid, 16. stearic acid, 17. elaidic acid, 18. oleic acid, 19. linolealidic acid, 20. linoleic acid, 21. arachidic acid, 22. linolenic acid, and 23. cis-11-eicosenoic acid. IS—internal standard.

The chromatographic conditions were previously optimized and validated for the GC-FID analysis of FA in cheese. Peaks were identified using retention times obtained from research carried out by the manufacturer on the standard mixture purchased from Supelco (37-component FAME mixture) with the column [17]. The FAMEs were separated according to the carbon number (the number of carbon atoms in the FA chain, excluding the methyl ester carbon) and the degree of unsaturation. Additionally, the position of the double bond(s) and the geometric configuration (cis/trans) are also important parameters. Their determination adds extra information to the characterization of the lipid fraction in cheese. In our study, 23 different FAs were separated and identified, ranging from short-chain (C4:0) to long-chain (C20:1n9) FAs. A good separation was obtained, except for the following compounds: cis- and trans-C18:1 (elaidic and oleic acids), which were coeluted. A typical chromatogram of FAMEs from cow cheese is shown in Figure 1.

GC-FID is one of the most robust techniques used to detect various FAs. MS-based detection (e.g., GC-MS) is a more sensitive alternative to FID, but FID is advantageous due to its stability, wide dynamic range, and introduction and maintenance costs. The FID signal is proportional to the number of carbon atoms in a hydrocarbon molecule. In the case of determining the FA percentage (in the wide FA range from C4:0 to C20:0), the results are riddled with errors. An additional factor that can contribute to the FID signal reduction is the presence of heteroatoms in a molecule. In the case of esters, a carbon atom is already oxidized in the starting sample. The oxidation energy leads to ionization, so in these molecules, the oxidized carbon atom is split, and this fragment cannot produce ions and a response in the detector. When this happens, it is necessary to use a response factor relative to each analyte with respect to an internal standard to correct the responses of the detector [13,18,19]. The experimental response factors (ERFs), theoretical response factors (TRFs), and error factors (EFs) used here were described in our previous study [13], and are listed in Table S1.

The precision of the quantitative methods was evaluated using intra-day repeatability and inter-day reproducibility experiments. Intra-day repeatability was determined from six complete analyses of each sample under the same conditions in one day. Inter-day reproducibility was determined from three complete analyses of each sample repeated on three consecutive days. Both intra-day and inter-day precision levels were satisfactory. The intra-day relative standard deviation (RSD) ranged from 0.4 to 3.8% and the inter-day RSD ranged from 0.5 to 7.6% (Table S1).

### 2.2. Comparison of Preparation Methods for FAME Determination

In the literature [20,21,22,23,24,25] and international standards [26], different methods have been proposed for the determination of FA profiles in cheese, mainly based on chromatographic analysis. Due to the ‘complexity’ of the matrix that is cheese, each procedure involves multiple steps. This can lead to analytical errors, resulting in a lack of reproducibility and unreliable results. Typically, the procedure for determining FA profiles in cheese follows three distinct steps: fat isolation, transesterification, and FA extraction. We selected several sample preparation methods that differed from each other to conduct our study, while the chromatographic analyses were carried out identically. One method that we did not use for our study is the one presented in ISO 14156, in which the fat is isolated using a Soxhlet extractor, because this method is very time-, labor-, and energy-intensive, and also involves considerable amounts of toxic solvents (more than 250 mL/sample). The Folch method, in which a chloroform–methanol mixture is used for fat isolation, is the most commonly applied procedure for FA determination. Many modifications have been introduced to this method to increase the efficiency of lipid isolation. Other methods of determining FA profiles in cheese are also used, in which safer solvents or solvent mixtures replace the toxic chloroform.

Our study selected six cheese sample preparation methods to determine the FA profile. The methods we chose varied in terms of the amount of reagents used, time consumption, and labor intensity. Three of these were the modified variants of the Folch method (D, E, and F). Each of the three Folch methods used different modifications to the original Folch method [27]. These modifications involved using different weighed amounts of cheese and different volumes of the chloroform–methanol mixture (2:1, *v*:*v*). In addition, in order to increase the efficiency of fat isolation, in Method E, an antioxidant (BHT) was added to the sample, and ultrasonication was applied, while in Method F, the resulting chloroform and aqueous layers were left for 24 h for more efficient/effective phase separation. In all the Folch-modified methods, the same amount of fat (100 mg) for the transesterification step was used. In the case of Method C, conversely to the Folch method, methanol and dichloromethane were used to isolate the fat, and all the extracted fat was trans-esterified. In addition, like Method E, Method C employed an antioxidant. The procedures of Methods A and B differed significantly from the others. It is presumed that their development aimed to identify a shortened/simplified and less solvent-intensive method of preparing cheese samples for analysis purposes. In Method A, the fat was not isolated from the cheese sample but was extracted directly with n-hexane, and the transesterification reaction was carried out at the same time. In contrast, in Method B, ammonia, ethanol, and n-hexane were added to the cheese sample in the first step, and ethanol and n-hexane were then added twice.

Table 1 shows the FA profiles produced using Methods A–F. Unfortunately, no comparable study results were obtained. This is most evident for Methods A, B, and C (*p* > 0.05). The percentage of short-chain FAs (C4:0–C11:0) was very low in Methods A and B, while their content was highest in Method C. However, it was noted that for Method C, the resolution of chromatographic peaks deteriorated after several chromatographic analyses (about twenty). This indicated that there was contamination in the chromatographic system; sediment was found in the liner, pre-column, and gold seal (Figure S2). For the other methods, no such contamination was observed. This problem may have been due to the difference in the fat isolation from the cheese sample. Namely, Method C used methanol and dichloromethane for fat isolation. In the final step of this procedure, a derivatization reagent was added to the total fat obtained. This may have influenced the poorly selective isolation of fat from the cheese sample, resulting in other matrix components, i.e., protein, passing into the extract.

The highest convergence for most acids (*p* < 0.05) was found for the three modified variants of the Folch method (D–F). Different modifications in the Folch method did not affect the differences in the FA profile of the cheese sample. Still, differences in the intensity of the chromatographic peaks could be observed. Compared to Method D, Methods E, and F produced higher-intensity FA peaks (Figure S1). In Method E, the higher peak intensity, or extraction efficiency, may have been due to the use of an antioxidant and ultrasonication. The additional sonication may have contributed to increased diffusion between the cheese sample and the chloroform–methanol mixture. In Method F, the increase in extraction efficiency may have been influenced by the fact that the mixture of aqueous and chloroform phases was left until the following day, resulting in an effective separation of the two.

Given the comparable results produced by the modified variants of the Folch method (D–F), it can be concluded that they were the most reliable. In addition, the results of the FAs content in the Gouda-type cheese sample obtained by modified Folch methods are similar to the data presented in the literature [28,29,30]. This confirms the use of the Folch method as a reference method. Of the three modification of the Folch methods investigated, Method D was the most favorable due to the lowest time consumption and the least amount of stages.

### 2.3. Assessment of the Method of Greenness

This study assessed the ‘green’ nature of the compared analytical methods for operators and the environment, including solvent characterization, experiment time, energy consumption, and others. Two different matrices were used, the Analytical Eco-Scale tool, which is based on scoring (numerical assessment), and the Analytical Greenness Metric for Sample Preparation (AGREEprep) tool, which combines a graphical representation of the results with scoring.

The Analytical Eco-Scale score was calculated by subtracting penalty points (PPs) from a base score of 100 for any factor in the analytical procedure, such as reagent quantity, hazard, energy consumption, and waste production. Green analysis was deemed ideal if it had an eco-scale value of 100, excellent if > 75, acceptable if > 50, and inadequate if < 50 [31,32].

The AGREEprep tool was first introduced in 2022 [16]. This metric tool gives prominence to the sample preparation step of the analysis. The AGREEprep tool includes software (free website link) that generates a pictogram showing the performance of the method [33]. This metric tool is based on ten effect categories, which are recalculated into sub-scores on a 0–1 scale. The color of each section ranges from red to green. The categories include, but are not limited to, hazardous reagent consumption, waste generation, sample volume, throughput, and energy consumption. The evaluation produces a pictogram that summarizes the overall greenness of the method. The different parts of the pictogram allow the identification of weak and strong points of the method and provide a quick comparison of different methods [16]. The evaluation results of the methods used to analyze the FA profiles are shown in Table 2.

The AGREEprep tool more rigorously evaluated the procedures used, with summary pictograms colored orange to red and numerical scores ranging from 0.13 to 0.27. It can be seen that the first higher-scored group of procedures comprised Methods A, B, and C, which received the same total score of 0.27. The second group of Methods—D, E, and F—received lower scores of 0.14 (for Methods D and E) and 0.13 for (Method F).

A more focused evaluation of the first three methods was mainly influenced by criteria 5 and 8, which relate to a smaller sample size (0.05–1 g) and lower energy consumption. However, Methods D–F used time-consuming and energy-intensive steps, i.e., homogenization, ultrasonication, and centrifugation. The other evaluation parameters are comparable for all methods. Detailed method evaluation reports using the AGREEprep tool are presented in the Supplementary Materials.

In the Analytical Eco-Scale assessment, all six methods scored ‘acceptable green analysis’ with scores ranging from 61 to 73. Method A received the highest score, while Methods D and F received the lowest score. Details of the input data used to assess each subcategory in Table S2 are provided in the Supplementary Materials.

The two scoring methods used to evaluate the methods used are not consistent with the authors’ assessment. Our observations clearly show that Method F was the least favorable in all respects. This assessment is due to the high workload of the analyst and the sample preparation time of about 24 h; in addition, the procedure consisted of the largest number of steps (about 20). Sample preparation required 155 mL of hazardous materials (n-hexane, methanol, and KOH), resulting in large amounts of waste. However, this parameter (criterion 2 for the AGREEprep tool) scored similarly across all procedures, ranging from 0.13 (A) to 0.0 (B–F). This is due to the fact that sample preparation methods that used more than 10 mL or 10 g of hazardous solvents and reagents had a score of 0 on this rule. Similarly, to assess the amount of waste generated (criterion 4), Method E generated approx. 26 mL of waste and received a score of 0.1, while Method F generated approx. 300 mL of waste and received a similar score of 0.0.

Therefore, it can be concluded that the greenness assessment methods of sample preparation for analysis purposes are not adequate for comparing (classic) techniques such as those presented in this study. Greenness assessment methods help compare micro-extraction, automated, solvent-free, or low-solvent consumption methods.

## 3. Materials and Methods

### 3.1. Cheese Sample

The experimental material was commercial Gouda cheese, produced using cow milk from a Polish producer (Pomerania, Poland). The cheese was cut into smaller sections, vacuum-packed, and frozen at −18 °C. All experiments were performed using a fresh-opened cheese sample after it was defrosted at room temperature and ground it into small pieces using a cheese grater. The same cheese sample was used for all methods examined in this article.

### 3.2. Chemical and Reagents

Solvents and chemicals used for lipid extraction and FAME preparation were of analytical grade. Chloroform, dichloromethane, methanol, n-hexane, ethanol, hydrochloric acid, ammonia, anhydrous sodium sulfate, butylated hydroxytoluene (BHT), potassium chloride, sodium chloride, potassium hydroxide, sodium hydroxide, and hexadecane (used as internal standards) were purchased from Sigma Aldrich (Darmstadt, Germany). The standard mix of 37 FAMEs and the standard mix of C4:0–C24:0 saturated FAMEs were purchased from Supelco (Bellefonte, PA, USA).

### 3.3. Lipid Extraction and FAME Preparation

In this study, six different sample preparation methods were tested. The procedures were carried out as follows:

- Method A [20]: 50 mg of cheese was mixed with 1 mL of n-hexane and 0.2 mL of KOH/MeOH (0.2 M). The sample was vortexed for 3 min and allowed to rest for 15 min, and 1 mL of HCl/MeOH (10%) was added. The sample was vortexed for 10 s and incubated (50 °C/10 min). After cooling, 2 mL of ultrapure water and 2 mL of n-hexane were added to the sample. The sample was mixed for 10 s and centrifuged for 5 min, and then a 0.5 g of anhydrous sodium sulfate was added. The sample was vortexed for 30 s and centrifuged for 5 min. The n-hexane phase was collected. The total time of sample preparation was about 60 min.
- Method B [21]: 1 g of cheese was added to 0.4 mL of ammonia (25%), 1 mL of EtOH (95%), and 5 mL of n-hexane. After centrifugation, the upper layer was collected and the sample was re-extracted with 1 mL of EtOH (95%) and 5 mL of n-hexane. The sample was centrifuged, the upper layer was collected, and the sample was extracted again using 5 mL of n-hexane. All the obtained phases were collected. The upper phases obtained during each extraction were pooled together, dried under nitrogen, and dissolved in 1 mL of n-hexane. The total time of sample preparation was about 60 min.
- Method C [22]: 1 g of cheese was mixed (250 rpm; 3 min) with 4 mL of MeOH, 2 mL of dichloromethane, and 1 mg of BHT. Then, 2 mL of dichloromethane and 2 mL of distilled water were added to the sample and gently shaken for 20 s, followed by centrifugation (2800 rpm; 15 min). The apolar layer was collected in a glass vial, and the sample was evaporated with nitrogen (40 °C; 25 min). Afterward, 100 µL of KOH/MeOH (0.2 M) was added to the sample. The mixture was incubated (95 °C; 20 min) and cooled to stop the derivatization reaction, and 1 mL of n-hexane was added. The total time of sample preparation was about 90 min.
- Method D [23]: 1 g of cheese was homogenized in 15 mL of chloroform–methanol (2:1; *v*/*v*). The mixture was shaken mechanically (20 min) and centrifuged (7300 rpm; 5 min). Then, the mixture was filtered, and then 15 mL of chloroform–methanol and 3 mL of KCl (0.74%) were added to the filtrates. After centrifugation (7300 rpm; 5 min), the chloroform layer was collected and mixed with 3 g of anhydrous sodium sulfate. Then, the mixture was filtrated and the extract was concentrated by removing chloroform in a rotary evaporator and dried over a gentle stream of nitrogen. Then, 100 mg of the obtained fat was weighed in a test tube and dissolved in 5 mL of n-hexane. Next, 0.2 mL of KOH/MeOH (0.2 M) was added to the mixture and shaken vigorously with a vortex mixer (1 min). After an additional reaction time of 5 min, 0.5 g of anhydrous sodium hydrogen sulfate was added and mixed again. The sample was centrifuged (3 min) and the extract was collected. The total time of sample preparation was about 95 min.
- Method E [24]: 2.5 g of cheese was added to a 25 mL of chloroform–methanol (2:1; *v*/*v*) and BHT (0.001%). The mixture was homogenized (2500 rpm; 30 min) and ultrasonicated (Amplifier 35%; 20 min), and 10 mL of saturated NaCl solution was added. The suspension was then centrifuged (20 min; 4000 rpm). The chloroform layer was removed using a rotary evaporator. Then, 100 mg of the obtained fat was weighed in a test tube and dissolved in 5 mL of n-hexane. Next, 0.2 mL of KOH/MeOH (0.2 M) was added to the mixture and shaken vigorously with a vortex mixer (1 min). After an additional reaction time of 5 min, 0.5 g of anhydrous sodium hydrogen sulfate was added and mixed again. The sample was centrifuged (3 min) and the extract was collected. The total time of sample preparation was about 100 min.
- Method F [25]: The 3 g samples were homogenized (1 min) with 30 mL of MeOH. Then, 30 mL of chloroform was added, and the mixture was homogenized (2 min). The prepared mixture was filtered into a glass cylinder. The solid residue was mixed in 60 mL of chloroform–methanol (2:1; *v*/*v*) and homogenized again for 3 min. The mixture was transferred to the same cylinder. Next, NaCl (0.88%) in water was added to the total filtrate (in the amount constituting ¼ of the filtrate volume), then shaken and left overnight. The lower layer was mixed with H_2_O/MeOH (1:1; *v*/*v*). The washing procedure was repeated. The remaining layer was dehydrated with anhydrous sodium sulfate and the mixture was evaporated. Then, 100 mg of the obtained fat was weighed in a test tube and dissolved in 5 mL of n-hexane. Next, 0.2 mL of KOH/MeOH was added to the mixture and shaken vigorously with a vortex mixer (1 min). After an additional reaction time of 5 min, 0.5 g of anhydrous sodium hydrogen sulfate was added and mixed again. The sample was centrifuged (3 min) and the extract was collected. The total time of sample preparation was about 26 h.

### 3.4. GC Analysis

The FA profile identification and quantification processes were performed using a gas chromatograph (Agilent 7890B, Santa Clara, CA, USA), equipped with a flame ionization detector (FID) and a fused silica capillary SP-2380 column (30 m × 0.25 mm × 0.2 µm) (Supelco, Bellefonte, PA, USA), with a constant flow of 1.0 mL/min helium as the carrier gas.

The injector port was held at 230 °C and used in the split mode using a split ratio of 10:1, and injection volumes were 1 µL. The detector temperature was 250 °C. The GC oven temperature program started at 50 °C and increased to 220 °C at 4 °C/min where it was held for 15 min.

### 3.5. Statistical Analysis

Peak areas were obtained by manual integral with Agilent ChemStation F.01.00.1903 (Agilent Technologies, Santa Clara, CA, USA). The experiments were performed in triplicate and the analysis was repeated at least three times. The results were expressed as the mean ± standard deviation. The data were subjected to a one-way analysis of variance (ANOVA) and Tukey’s test. Results were considered statistically significant at *p* < 0.05.

## 4. Conclusions

The methodologies chosen to extract the lipid fraction from the cheese matrix may differently affect the yield of extracted lipids. In this study, six analytical methods were used to determine the FA profile of the cheese sample. In order to simplify the selection of the method used for the routine analyses of the FA profile in cheese, an assessment of the environmental impact of these methods was used. To this end, the Analytical Eco-Scale tool and the Analytical Greenness Metric for Sample Preparation (AGREEprep) tool were applied.

The results showed significant differences (*p* > 0.05) in the content of the individual acids obtained using the different methods. It was observed that the results generated by the three modified Folch method variants showed the highest similarity and the least significant differences (*p* < 0.05). In contrast, the other three methods showed a large discrepancy between the results (*p* > 0.05). However, Methods A, B, and C obtained better scores on the assessment of greenness than the modified Folch methods.

This study shows that it is important to compare different methodologies for the determination of fatty acids in complex matrices such as cheese. The use of greenness tools allows the suitability of these analytical procedures to be assessed in terms of environmental friendliness. In addition, an assessment of analytical methods results in significant economic benefits (costs of reagents and waste, time and energy reductions, etc.) and will positively affect the health of operators.

## Figures and Tables

**Table 1:** Contents of fatty acids determined in cheese using different sample preparation methods (mean values in g/100 g FA ± SD).

Nr of FA	FA	Method A	Method B	Method C	Method D	Method E	Method F
1	C4:0	0.01 ± 0.01 ^a^	0.06 ± 0.01 ^a^	4.80 ± 0.72 ^c^	2.72 ± 0.24 ^b^	2.97 ± 0.27 ^b^	2.87 ± 0.39 ^b^
2	C6:0	0.01 ± 0.00 ^a^	0.09 ± 0.02 ^a^	1.93 ± 0.29 ^b^	1.41 ± 0.31 ^bc^	1.13 ± 0.34 ^c^	1.23 ± 0.39 ^c^
3	C8:0	0.05 ± 0.04 ^a^	0.27 ± 0.01 ^a^	1.54 ± 0.10 ^c^	1.08 ± 0.20 ^b^	0.81 ± 0.23 ^b^	0.90 ± 0.36 ^b^
4	C10:0	1.06 ± 0.15 ^a^	0.51 ± 0.21 ^a^	4.66 ± 0.13 ^d^	2.70 ± 0.09 ^b^	2.43 ± 0.21 ^bc^	2.01 ± 0.52 ^c^
5	C11:0	0.01 ± 0.00 ^c^	0.11 ± 0.03 ^ab^	0.11 ± 0.01 ^ab^	0.10 ± 0.00 ^ab^	0.07 ± 0.01 ^a^	0.12 ± 0.05 ^a^
6	C12:0	2.96 ± 0.18 ^a^	1.42 ± 0.30 ^d^	4.98 ± 0.13 ^e^	3.91 ± 0.21 ^b^	3.47 ± 0.24 ^abc^	2.94 ± 0.53 ^ac^
7	C13:0	0.10 ± 0.00 ^a^	0.49 ± 0.13 ^b^	0.16 ± 0.01 ^a^	0.15 ± 0.01 ^a^	0.12 ± 0.01 ^a^	0.12 ± 0.03 ^a^
8	C14:0	12.07 ± 0.40 ^a^	8.83 ± 0.86 ^d^	13.94 ± 0.30 ^e^	12.17 ± 0.58 ^ab^	11.68 ± 0.45 ^abc^	11.82 ± 1.54 ^abc^
9	C14:1	1.20 ± 0.06 ^ab^	0.96 ± 0.14 ^ac^	1.75 ± 0.06 ^d^	1.39 ± 0.07 ^bde^	1.30 ± 0.04 ^bcef^	1.30 ± 0.33 ^bcef^
10	C15:0	1.95 ± 0.14 ^a^	1.43 ± 0.04 ^f^	2.21 ± 0.17 ^abc^	2.28 ± 0.09 ^cd^	1.96 ± 0.04 ^be^	2.02 ± 0.28 ^bde^
11	C15:1	0.41 ± 0.02 ^a^	0.78 ± 0.36 ^b^	0.51 ± 0.29 ^abc^	0.37 ± 0.01 ^ac^	0.32 ± 0.00 ^ac^	0.36 ± 0.08 ^ac^
12	C16:0	43.85 ± 1.33 ^a^	37.17 ± 0.23 ^b^	38.17 ± 1.22 ^b^	41.62 ± 1.12 ^ac^	42.45 ± 0.63 ^ac^	41.94 ± 1.87 ^ac^
13	C16:1	2.15 ± 0.10 ^ab^	2.56 ± 0.03 ^c^	2.21 ± 0.09 ^ad^	2.42 ± 0.07 ^c^	2.18 ± 0.03 ^bde^	2.15 ± 0.12 ^bde^
14	C17:0	0.98 ± 0.06 ^a^	0.86 ± 0.12 ^a^	0.90 ± 0.20 ^a^	0.99 ± 0.03 ^a^	0.93 ± 0.02 ^a^	0.98 ± 0.12 ^a^
15	C17:1	0.45 ± 0.01 ^a^	0.70 ± 0.16 ^b^	0.57 ± 0.24 ^abc^	0.46 ± 0.05 ^acd^	0.41 ± 0.01 ^acde^	0.48 ± 0.06 ^acde^
16	C18:0	8.29 ± 0.66 ^b^	9.00 ± 1.35 ^b^	4.56 ± 0.09 ^c^	6.41 ± 0.28 ^a^	6.62 ± 0.24 ^a^	6.47 ± 0.29 ^a^
17 + 18	C18:1n9t + C18:1n9c	19.60 ± 1.46 ^b^	23.80 ± 0.59 ^c^	12.76 ± 0.16 ^d^	15.43 ± 1.12 ^a^	16.66 ± 0.56 ^a^	15.36 ± 1.67 ^a^
19	C18:2n6c	0.65 ± 0.05 ^a^	0.93 ± 0.33 ^b^	0.54 ± 0.06 ^a^	0.71 ± 0.02 ^ab^	0.67 ± 0.04 ^a^	0.45 ± 0.37 ^a^
20	C18:2n6t	1.99 ± 0.08 ^a^	2.67 ± 0.23 ^d^	1.46 ± 0.04 ^b^	1.68 ± 0.07 ^bc^	1.65 ± 0.05 ^bc^	1.75 ± 0.26 ^ac^
21	C20:0	0.17 ± 0.02 ^b^	0.28 ± 0.07 ^a^	0.28 ± 0.09 ^a^	0.28 ± 0.01 ^a^	0.23 ± 0.02 ^a^	0.21 ± 0.08 ^a^
22	C18:3n3	1.11 ± 0.08 ^a^	5.24 ± 0.68 ^b^	1.03 ± 0.06 ^a^	1.07 ± 0.04 ^a^	1.11 ± 0.04 ^a^	1.11 ± 0.07 ^a^
23	C20:1n9	0.92 ± 0.02 ^a^	1.84 ± 0.31 ^c^	0.92 ± 0.07 ^a^	1.12 ± 0.05 ^ab^	1.13 ± 0.05 ^ab^	1.23 ± 0.17 ^b^
Sums							
∑SFA ^1^		71.52	60.53	78.25	75.82	74.87	73.62
∑UFA ^2^		24.73	30.64	18.71	21.19	21.99	20.88
∑MUFA ^3^		3.74	8.84	3.04	3.46	3.43	3.31
∑PUFA ^4^		20.25	24.73	13.30	16.15	17.32	15.80

^1^ ∑SFA = sum of saturated fatty acids; ^2^ ∑UFA = sum of unsaturated fatty acids; ^3^ ∑MUFA = sum of monounsaturated fatty acids; ^4^ ∑PUFA = sum of polyunsaturated fatty acids. Mean values with similar letters within a row are statistically similar, while mean values with different letters within a row are significantly different (*p* < 0.05).

**Table 2:** The greenness profile of the employed (A–F) methods for FA analysis in cheese samples using Eco-Scale and AGREE-prep metrics.

Method	Metrics	
	Analytical Eco-Scale Score	AGREEprep Pictogram
A	73 acceptable green analysis	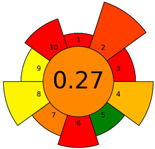
B	63 acceptable green analysis	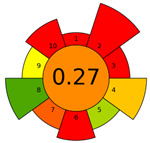
C	71 acceptable green analysis	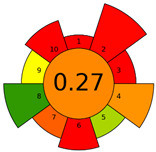
D	61 acceptable green analysis	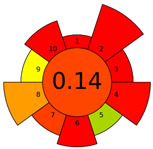
E	63 acceptable green analysis	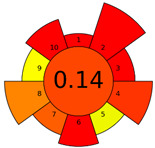
F	61 acceptable green analysis	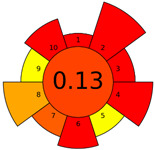

## Data Availability

Data is contained within the article or Supplementary Material.

## Supplementary Materials

The following supporting information can be downloaded at: https://www.mdpi.com/article/10.3390/molecules28134981/s1. Figure S1: Chromatograms obtained Methods A, B, C, D, E, and F. Figure S2: Inlet gold seal and inlet liner after chromatographic analysis of samples (about 20) made using Method C. Table S1: Experimental and theoretical correction factors, error factor, and intra-day and inter-day precision for the fatty acid in cheese sample. Table S2: Calculated PPs (Eco-Scale) for evaluated analytical procedures for FA determination in cheese samples (Methods A–F). Reports from the AGREEprep tool for Methods A–F.
