# Supplementary material for: A Comparative Analysis on the Environmental Impact of Selected Methods for Determining the Profile of Fatty Acids in Cheese
Source: Molecules. 2023 Jun 25;28(13):4981. doi: 10.3390/molecules28134981 (PMC10343173; doi:10.3390/molecules28134981)
Supplement: Supplementary file 1 [file molecules-28-04981-s001.zip › molecules-2453735-supplementary.pdf]

Supplementary Materials

A Comparative Analysis on the Environmental Impact of Selected Methods for Determining the Profile of Fatty Acids in Cheese

Table S1. Experimental and theoretical correction factors, error factor, intra-day and inter-day precision for the fatty acid in cheese sample.

Fatty acid		ERF [1]	TRF [2]	EF [3]	Intra-day RSD (%)	Inter-day RSD (%)
butyric acid	C4:0	1.2144	1.5742	0.7714	3.8	3.3
caproic acid	C6:0	1.0371	1.3378	0.7752	3.1	1.0
caprylic acid	C8:0	1.0509	1.2195	0.8617	2.9	1.9
capric acid	C10:0	1.0681	1.2702	0.8408	1.4	1.5
undecanoic acid	C11:0	1.0552	1.1486	0.9186	2.6	1.6
lauric acid	C12:0	1.0614	1.1013	0.9637	2.3	3.7
oleic acid	C13:0	1.0516	1.0831	0.9709	3.1	4.0
myristic acid	C14:0	1.0432	1.0675	0.9772	2.1	3.9
myristoleic acid	C14:1	1.0372	1.0587	0.9796	2.5	5.1
pentadecylic acid	C15:0	1.0226	1.0540	0.9702	2.6	2.6
ginkgolic acid	C15:1	1.0211	1.0457	0.9764	2.3	7.6
palmitic acid	C16:0	0.9816	1.0422	0.9418	2.2	3.3
palmitoleic acid	C16:1	1.0008	1.0345	0.9674	1.7	1.3
heptadecanoic acid	C17:0	0.9920	1.0318	0.9614	0.3	2.1
10-heptadecenoic acid	C17:1	0.9953	1.0244	0.9715	0.6	3.3
stearic acid	C18:0	0.9659	1.0225	0.9446	0.8	2.1
elaidic acid + oleic acid	C18:1n9t + C18:1n9c	0.9630	1.0155	0.9483	0.9	2.0
linolealidic acid	C18:2n6c	0.9814	1.0087	0.9729	0.7	2.2
linoleic acid	C18:2n6t	0.9883	1.0087	0.9797	0.9	2.5
arachidic acid	C20:0	0.9813	1.0067	0.9747	0.5	2.0
alpha-linolenic acid	C18:3n3	0.9821	1.0017	0.9804	0.4	0.5
11-eicosenoic acid	C20:1n9	1.4001	1.0005	1.3994	1.9	1.5

[1] ERF = Experimental response factor, [2] TRF = Theoretical response factor, [3] EF = Error factor (ERF/TRF).

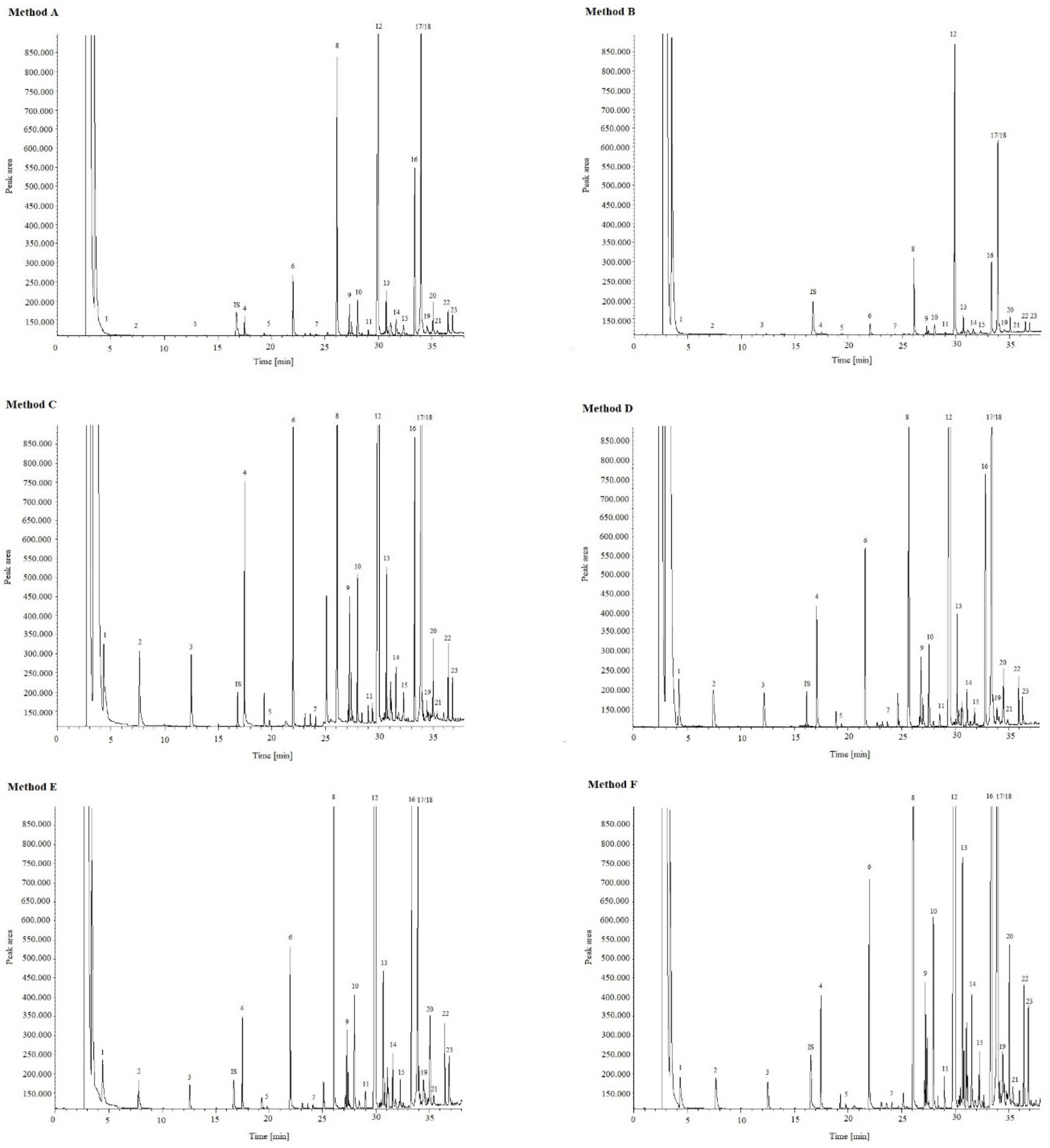

Figure S1. Chromatograms obtained A, B, C, D, E, and F methods.

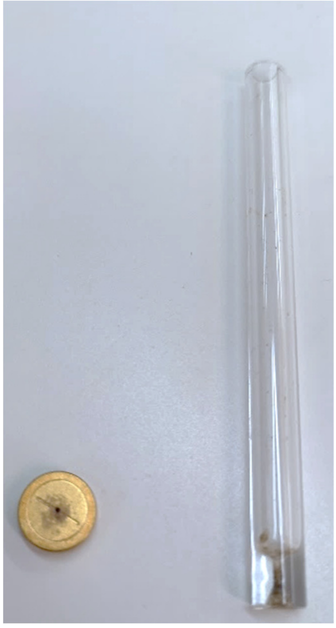

Figure S2. Inlet gold seal and inlet liner after chromatographic analysis of samples (about 20) made using the C method.

Table S2. Calculated PPs (Eco-Scale) for evaluated analytical procedures for FAs determination in cheese samples (Procedures A - C).

**Procedure A**		**Procedure B**		**Procedure C**	
**Reagents**	**PPs**	**Reagents**	**PPs**	**Reagents**	**PPs**
Methanol: 0.1 mL	6	Ethanol: 2 mL	2	Methanol: 4.1 mL	6
Na_2SO_4(aq.): 0.5 g	0	*n*-hexan: 16 mL	16	KOH: 0.05 g	2
KOH: 0.1 g	2	NH_3 (aq.): 0.4 mL	6	*n*-hexan: 1 mL	8
n-hexan: 4 mL	8			Dichloromethane: 4 mL	2
	∑16		∑ 24		∑ 18
**Instrument**	**PPs**	**Instrument**	**PPs**	**Instrument**	**PPs**
Transport	1	Transport	1	Transport	1
Energy (≤0.1 kWh)	0	Energy (≤0.1 kWh)	0	Energy (≤0.1 kWh)	0
Occupational hazard	3	Occupational hazard	3	Occupational hazard	3
GC-FID	1	GC-FID	1	GC-FID	1
Waste (1-10 mL, no treatment)	6	Waste (>10 mL, no treatment)	8	Waste (1-10 mL, no treatment)	6
	∑11		∑13		∑11
**Total PPs**	**27**	**Total PPs**	**37**	**Total PPs**	**29**
**Score – acceptable green analysis**	**73**	**Score – acceptable green analysis**	**63**	**Score – acceptable green analysis**	**71**

Table S2 con. Calculated PPs (Eco-Scale) for evaluated analytical procedures for FAs determination in cheese samples (Procedures D - F).

**Procedure D**		**Procedure E**		**Procedure F**	
**Reagents**	**PPs**	**Reagents**	**PPs**	**Reagents**	**PPs**
Chloroform: 20 mL	4	Chloroform: 16 mL	4	Methanol: 80 mL	12
Methanol: 10.1 mL	12	Methanol: 11 mL	12	Chloroform: 70 mL	4
Na_2SO_4: 3.5 g	0	NCl (aq): 15 mL	0	NCl (aq): 30 mL	0
KCl (o.74%): 3 g	0	NaOH: 0.2 g	2	Na_2SO_4: 0.5 g	0
n-hexan: 5 mL	8	Isooctane: 3.5 mL	8	KOH: 0.05 g	2
KOH: 0.1 g	2	Na_2SO_4: 2 g	0	*n*-hexan: 5 mL	8
	∑26		∑ 26		∑ 26
**Instrument**	**PPs**	**Instrument**	**PPs**	**Instrument**	**PPs**
Transport	1	Transport	1	Transport	1
Energy (≤0.1 kWh)	0	Energy (≤1.5 kWh)	1	Energy (≤0.1 kWh)	0
Occupational hazard	3	Occupational hazard	0	Occupational hazard	3
GC-FID	1	GC-FID	1	GC-FID	1
Waste (>10 mL, no treatment)	8	Waste (>10 mL, no treatment)	8	Waste (>10 mL, no treatment)	8
	∑13		∑11		∑13
**Total PPs**	**39**	**Total PPs**	**37**	**Total PPs**	**39**
**Score – acceptable green analysis**	**61**	**Score – acceptable green analysis**	**63**	**Score – acceptable green analysis**	**61**

AGREEprep
Analytical Greenness Metric for
Sample Preparation

08/05/2023 21:24:17

Method A

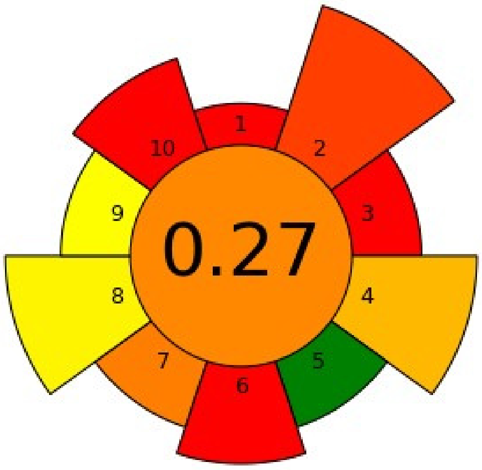

#	Criterion	Score	Weight
1.	**Sample preparation placement**<br>Sample preparation placement: Ex situ	0.0	1
2.	**Hazardous materials**<br>Mass [g] or volume [mL] of problematic materials: 4.2	0.13	5
3.	**Sustainability and renewability of materials**<br>< 25% of reagents and materials are sustainable or renewable and can only be used once	0.0	2
4.	**Waste**<br>Mass [g] or volume [mL] of waste: 5.2	0.36	4
5.	**Size economy of the sample**<br>Mass [g] or volume [mL] of the sample: 0.05	1.0	2
6.	**Sample throughput**<br>Hourly sample throughput: 1	0.0	3
7.	**Integration and automation**<br>No. of sample prep. steps: 2 steps or fewer; degree if automation: Manual systems	0.25	2

8.	**Energy consumption**<br>Approximate energy consumption per analysis [W]: 76	0.48	4
9.	**Post-sample preparation configuration for analysis**<br>GC with non-MS detection, atomic absorption spectroscopy, capillary electrophoresis, etc.	0.5	2
10.	**Operator's safety**<br>No. of distinct hazards: 4 or more hazards	0.0	3

AGREEprep
Analytical Greenness Metric for
Sample Preparation

09/05/2023 20:30:59

Method B

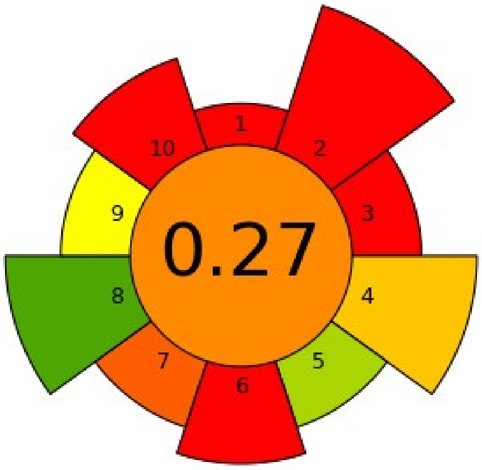

#	Criterion	Score	Weight
1.	**Sample preparation placement**<br>Sample preparation placement: Ex situ	0.0	1
2.	**Hazardous materials**<br>Mass [g] or volume [mL] of problematic materials: 18.4	0.0	5
3.	**Sustainability and renewability of materials**<br>< 25% of reagents and materials are sustainable or renewable and can only be used once	0.0	2
4.	**Waste**<br>Mass [g] or volume [mL] of waste: 4.4	0.39	4
5.	**Size economy of the sample**<br>Mass [g] or volume [mL] of the sample: 1	0.67	2
6.	**Sample throughput**<br>Hourly sample throughput: 1	0.0	3
7.	**Integration and automation**<br>No. of sample prep. steps: 3 steps; degree if automation: Manual systems	0.19	2

8.	**Energy consumption**<br>Approximate energy consumption per analysis [W]: 18	0.85	4

9.	**Post-sample preparation configuration for analysis**<br>GC with non-MS detection, atomic absorption spectroscopy, capillary electrophoresis, etc.	0.5	2

10.	**Operator's safety**<br>No. of distinct hazards: 4 or more hazards	0.0	3

AGREEprep
Analytical Greenness Metric for
Sample Preparation

09/05/2023 21:22:42

Method C

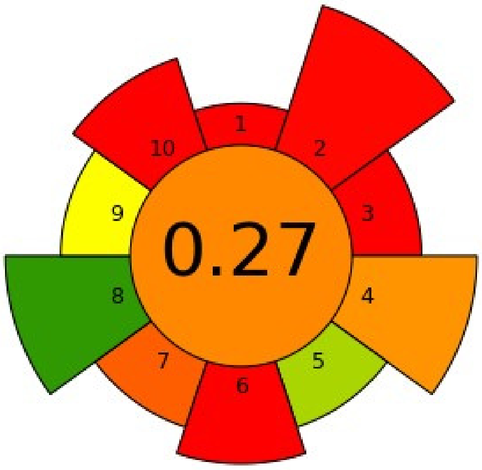

#	Criterion	Score	Weight
1.	**Sample preparation placement**<br>Sample preparation placement: Ex situ	0.0	1
2.	**Hazardous materials**<br>Mass [g] or volume [mL] of problematic materials: 9.1	0.01	5
3.	**Sustainability and renewability of materials**<br>< 25% of reagents and materials are sustainable or renewable and can only be used once	0.0	2
4.	**Waste**<br>Mass [g] or volume [mL] of waste: 8.1	0.29	4
5.	**Size economy of the sample**<br>Mass [g] or volume [mL] of the sample: 1	0.67	2
6.	**Sample throughput**<br>Hourly sample throughput: 0.66	0.0	3
7.	**Integration and automation**<br>No. of sample prep. steps: 3 steps; degree if automation: Manual systems	0.19	2

8.	**Energy consumption**	0.91	4
	Approximate energy consumption per analysis [W]: 14.3		

9.	**Post-sample preparation configuration for analysis**	0.5	2
	GC with non-MS detection, atomic absorption spectroscopy, capillary electrophoresis, etc.		

10.	**Operator's safety**	0.0	3
	No. of distinct hazards: 4 or more hazards		

AGREEprep
Analytical Greenness Metric for
Sample Preparation

09/05/2023 21:47:29

Method D

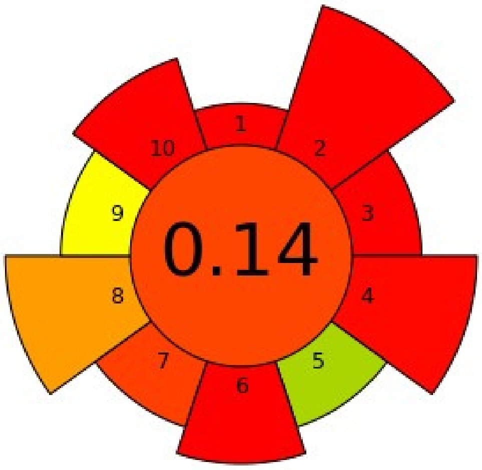

#	Criterion	Score	Weight
1.	**Sample preparation placement**<br>Sample preparation placement: Ex situ	0.0	1
2.	**Hazardous materials**<br>Mass [g] or volume [mL] of problematic materials: 35.2	0.0	5
3.	**Sustainability and renewability of materials**<br>< 25% of reagents and materials are sustainable or renewable and can only be used once	0.0	2
4.	**Waste**<br>Mass [g] or volume [mL] of waste: 44.7	0.02	4
5.	**Size economy of the sample**<br>Mass [g] or volume [mL] of the sample: 1	0.67	2
6.	**Sample throughput**<br>Hourly sample throughput: 0.6	0.0	3
7.	**Integration and automation**<br>No. of sample prep. steps: 4 steps; degree if automation: Manual systems	0.12	2

8.	**Energy consumption**	0.31	4
	Approximate energy consumption per analysis [W]: 148		

9.	**Post-sample preparation configuration for analysis**	0.5	2
	GC with non-MS detection, atomic absorption spectroscopy, capillary electrophoresis, etc.		

10.	**Operator's safety**	0.0	3
	No. of distinct hazards: 4 or more hazards		

AGREEprep
Analytical Greenness Metric for
Sample Preparation

10/05/2023 14:03:41

Method E

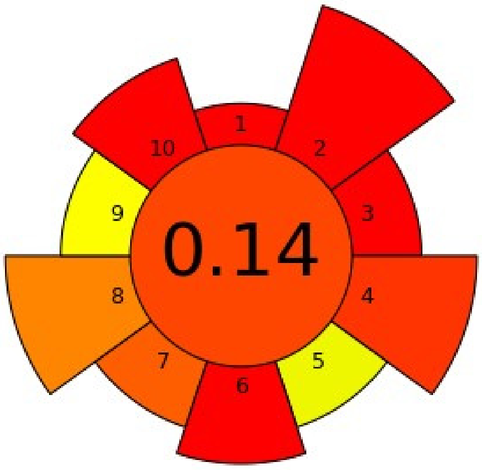

#	Criterion	Score	Weight
1.	**Sample preparation placement**<br>Sample preparation placement: Ex situ	0.0	1
2.	**Hazardous materials**<br>Mass [g] or volume [mL] of problematic materials: 35.2	0.0	5
3.	**Sustainability and renewability of materials**<br>< 25% of reagents and materials are sustainable or renewable and can only be used once	0.0	2
4.	**Waste**<br>Mass [g] or volume [mL] of waste: 26.2	0.1	4
5.	**Size economy of the sample**<br>Mass [g] or volume [mL] of the sample: 2.5	0.53	2
6.	**Sample throughput**<br>Hourly sample throughput: 0.6	0.0	3
7.	**Integration and automation**<br>No. of sample prep. steps: 3 steps; degree if automation: Manual systems	0.19	2

8.	**Energy consumption**<br>Approximate energy consumption per analysis [W]: 178	0.26	4

9.	**Post-sample preparation configuration for analysis**<br>GC with non-MS detection, atomic absorption spectroscopy, capillary electrophoresis, etc.	0.5	2

10.	**Operator's safety**<br>No. of distinct hazards: 4 or more hazards	0.0	3

AGREEprep
Analytical Greenness Metric for
Sample Preparation

10/05/2023 14:07:58

Method F

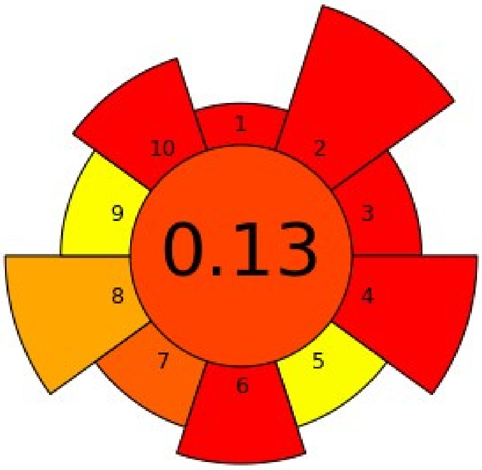

#	Criterion	Score	Weight
1.	**Sample preparation placement**<br>Sample preparation placement: Ex situ	0.0	1
2.	**Hazardous materials**<br>Mass [g] or volume [mL] of problematic materials: 195.2	0.0	5
3.	**Sustainability and renewability of materials**<br>< 25% of reagents and materials are sustainable or renewable and can only be used once	0.0	2
4.	**Waste**<br>Mass [g] or volume [mL] of waste: 301.7	0.0	4
5.	**Size economy of the sample**<br>Mass [g] or volume [mL] of the sample: 3	0.51	2
6.	**Sample throughput**<br>Hourly sample throughput: 0.04	0.0	3
7.	**Integration and automation**<br>No. of sample prep. steps: 3 steps; degree if automation: Manual systems	0.19	2

8.	**Energy consumption**<br>Approximate energy consumption per analysis [W]: 138	0.33	4

9.	**Post-sample preparation configuration for analysis**<br>GC with non-MS detection, atomic absorption spectroscopy, capillary electrophoresis, etc.	0.5	2

10.	**Operator's safety**<br>No. of distinct hazards: 4 or more hazards	0.0	3
